# Female Sex Workers, Male Circumcision and HIV: A Qualitative Study of Their Understanding, Experience, and HIV Risk in Zambia

**DOI:** 10.1371/journal.pone.0053809

**Published:** 2013-01-17

**Authors:** Sharon A. Abbott, Nicole A. Haberland, Drosin M. Mulenga, Paul C. Hewett

**Affiliations:** 1 Population Council, New York, New York, United States of America; 2 Population Council, Lusaka, Zambia; Yale School of Public Health, United States of America

## Abstract

Several sub-Saharan African countries, including Zambia, have initiated national voluntary medical male circumcision (MC) programs to reduce HIV incidence. In-depth interviews were conducted with twenty female sex workers (FSWs) in Lusaka to examine their understanding of MC and experiences with circumcised clients. Knowledge of MC was derived primarily through informal sources, with very few FSWs reporting exposure to MC educational campaigns. MC was not widely believed to be protective against HIV, however it was viewed by some as protective against STIs. Three FSWs reported having sex with recently circumcised clients, and most reported that men often used their MC status to try to convince FSWs to forego condoms. Findings suggest that FSWs, already at high risk for HIV infection, may face additional pressure toward higher risk behavior as a result of MC. As MC services are expanded, programs should support FSWs' efforts to protect themselves by providing information about what MC can - and cannot - offer for HIV/STI infection prevention.

## Introduction

Three randomized controlled trials (RCT) of male circumcision (MC) conducted in South Africa, Kenya and Uganda demonstrated that MC significantly reduces the risk of female to male HIV infection by about 60 percent [1]–[3]. In response, in 2007, WHO recommended that countries with generalized HIV epidemics and low MC prevalence expand access to MC services [4]. Encouraged by these results and recommendations, several countries in sub-Saharan Africa, including Zambia, have initiated national voluntary medical MC programs. In late 2009, the Zambian National Male Circumcision Strategy and Implementation Plan 2010–2020 was adopted by the Ministry of Health [5]; the plan was updated in 2012. The aims of the plan are to increase the number of sites providing MC services and increase the number of HIV-negative males 15–49 obtaining circumcision, with a target MC prevalence of 80% by 2015 [6].

The impact of an MC intervention on HIV infections averted, however, is considerably lowered if MC does not reach a significant proportion of the male population, or if risk-compensation post-circumcision occurs among men and their partners [7], [8]. Risk compensation occurs when the perceived reduction in HIV risk from MC leads to changes in behavior, including reduced condom use, increased numbers of sexual partners, decreased age of sexual debut, or other risky sexual behavior. To-date, however, there is scant evidence of risk compensation post-MC. A five year follow-up study of the RCT conducted in Uganda found no differences in the reported sexual behaviors of the circumcised men and control group [9]. However, a small qualitative study conducted in Swaziland found a slight increase in sexual risk taking and experimentation in the period following MC among a minority of men [10].

The number of HIV infections averted may also be lowered if a significant proportion of men who have obtained MC resume sex prior to wound healing. A Ugandan RCT among asymptomatic HIV-infected men found an increase in HIV risk for women whose recently circumcised partners resumed sex prior to wound healing, although the results were statistically significant at the p = .06 level [11]. In a study of MC clients in Kenya, 31% reported engaging in sexual activities 3–4 weeks post circumcision, substantially before the recommended six week post-surgical abstinence period [12]. A second study conducted in Zambia found that 24% of MC clients resumed sex before the recommended healing period; among MC clients who had ever had sex this figure was closer to one-third (30%). Further, among the men resuming sex early, 22% had sex within the first week post MC. The authors also found that risky behavior at baseline – including unprotected sex and number of partners – were good predictors of the early resumption of sex [13].

Little published research has been conducted with the female sexual partners of recently circumcised men, including female sex workers (FSWs). FSWs are of particular interest for understanding risk compensation and HIV transmission for several reasons. They are exposed to more sexually active men, they have to negotiate condom use with clients, and their clients are, by definition, engaged in higher risk behavior. In addition, as a vulnerable, at-risk and stigmatized population, it is important to assess the degree to which FSWs receive and understand HIV prevention messaging. This is no less the case with MC, particularly given the need to convey the complex message that MC is only partially protective for men, and that it does not provide direct protection from HIV for women [11].

Sex workers in Zambia, as in most African countries, carry a disproportionate burden in regard to HIV and AIDS. Studies conducted with FSWs in Southern Africa have found HIV prevalence rates 10–20 times higher among FSWs than among adults in the general population, with rates of HIV infection reaching 50% of all FSWs tested [14]. In a 2005 Biological and Behavioral Survey conducted in Ndola, Zambia, 65.4% of FSWs sampled were HIV- infected [15]. Female sex workers, along with their clients, play a crucial role in the spread of the virus to the general population [16]–[18]. Unprotected sex (sex without a condom) often results from refusal by a client and/or client brutality [14]. Women's health advocates are concerned that MC could further limit FSWs ability to negotiate condom use [19].

Sex workers in Zambia are a critical population for assessing behavior post-MC for several reasons. FSWs in Zambia report a high number of sexual partners (37% of FSWs report 20 or more sexual partners in the last 30 days) [20], and are likely to have been exposed to men who have been circumcised. In a 2009 survey, 51% of FSWs reported that their last sexual partner was circumcised [20]. Further, men report higher rates of condom use with FSWs compared with their primary intimate partners; 42% report using condom at last paid sex compared to 6% with an intimate partner [21]. This study examines the extent to which MC is understood by FSWs in Zambia and the impact of MC status on sexual negotiation, including the use of condoms, with the goal of informing HIV prevention programs reaching FSWs and advising behavioral change communication in the context of MC scale-up.

## Methods

### Study Locales and Interview Methods

The study was conducted among a convenience sample of 20 FSWs in Lusaka, Zambia. Lusaka, the capital of Zambia and its largest urban center, was selected as the location for this study because MC scale-up efforts were significantly underway at the time data collection began.

In-depth interviews (IDIs) were conducted between July and November 2010. Respondents were recruited from known “hot spots” — areas where FSWs and clients are known to meet. The hot spots included bars, nightclubs, brothels, and selected street locations. Respondents were approached during times in which they were available for sex work, but were not interacting with potential sex partners. An extensive review of the literature of FSWs in Africa conducted by Scorgie and colleagues (2011) found that venues frequented by sex workers are linked to their social status and hierarchies of income [14]. Efforts were made to stratify the selection of sex workers whose paying clients were assumed to be of a lower, middle and higher socioeconomic status.

Interviews were conducted by two trained female interviewers, both Zambian nationals. Interviewers were accompanied by a male escort familiar with the area, whose presence provided security and protection; the driver of the study vehicle was also male. The interviewers approached women they assumed to be FSWs based on dress, location, and time of day. After confirming that the woman self-identified as a sex worker and was at least 18 years old, the interviewers explained the purpose of the study and asked if she would be interested in participating. Interviews were typically one hour and were either conducted at the time of contact or appointments were made for a later date. Those interviews conducted “on the spot” took place in a variety of settings, including bars, a brothel, a private home, and in the study vehicle. For interviews conducted in the study vehicle, the driver and escort remained outside during the interview. Scheduled interviews were conducted in a variety of places such as a private office at the Population Council or a respondent's home. Interview settings were arranged based on the FSW's preference, with the objective of maintaining the privacy and confidentiality of the respondents.

A semi-structured interview guide addressed four primary themes – (1) background information (e.g., age, marital status, number of children and/or dependents, alcohol and drug use, experience of violence (sexual, physical, and emotional) by intimate partners, clients, and strangers), (2) condom use and negotiation with paying clients and nonpaying partners, (3) knowledge of MC, and sources of information about MC, and (4) sexual experiences with circumcised and uncircumcised sexual partners. The guide was designed to allow interviewers to pursue other lines of inquiry that emerged during the conversation.

All interviews were conducted in Nyanja, Bemba or English depending on the preference of the participant. Interviews were tape-recorded, transcribed and, if necessary, translated into English. Transcripts were examined by the authors through an iterative process to identify recurrent themes and to develop codes to be applied to the text. Themes were based on an inductive and deductive process from issues emerging from the data. Verbatim quotes from the data are used to support the presentation of results. All participants' identifying information has been removed to maintain the anonymity of the respondent.

### Ethical Considerations

The University of Zambia (UNZA) Biomedical Research Ethics Committee (Lusaka, Zambia), the Zambian Ministry of Health, and the Population Council Institutional Review Board (New York, USA) reviewed and approved the study protocol.

Standard ethical procedures for informed consent and confidentiality were administered before the IDIs were conducted. Prior to the IDI, respondents were informed of the purpose of the study and the voluntary nature of information given, and were required to sign the informed consent form; they could elect to sign with an X if they could not write. Based on UNZA-BREC recommendation, participants received refreshments and compensation for their time during the interview in the amount of 30,000 Kwacha (approximately $6 USD). At the end of the IDI, FSWs were given a fact sheet about MC and referral information for HIV testing and other health services and were offered male and female condoms.

## Results

### Participant Profiles

The twenty participants were between the ages of 19 and 35 (mean age 26.8 years old). Seventeen were Zambian nationals, and three were Zimbabwean. The three respondents from Zimbabwe worked in brothels, while the majority of Zambian FSWs found their clients in bars, nightclubs, or on the streets. Four of the FSWs were recruited from areas frequented by assumed lower income clients, twelve from areas with assumed middle income clients, and four from areas with assumed higher income clients. Almost all reported having children and/or dependents; typically, they were the sole financial provider for their families. Thirteen respondents had boyfriends or husbands, and of those, seven reported that their steady partners were married to someone else.

Similar to other studies of FSWs in Africa [14], with the exception of those working in brothels, most FSWs in this sample were operating without intermediaries. FSWs directly negotiated sexual acts to be performed, including sex with or without a condom, and payment with potential clients. They also handled all financial transactions themselves, which occasionally led to being robbed by clients and others; experiences of violence or threats of violence were common. Almost all of the participants (17 out of the 20 interviewed) reported experiencing physical or sexual abuse, as well as intimidation or threats. The use of alcohol to facilitate participants doing sex work was a dominant theme in the interviews. Half of the respondents reported that they were intoxicated most, if not all, of the time they were with clients

### The Context of Sex for FSWs

Our data suggest that both individual and structural-environmental factors place FSWs in this study at increased risk for HIV infection. Reports of unprotected sex with clients and non-paying partners and accounts of sexual encounters with men who appear to have STIs highlight the difficulties FSWs face – both routinely and in confronting men's risk compensation post-MC.

Half of the sample reported having encounters with clients who appeared to be suffering from sexually transmitted infections (STI) or what was assumed to be the signs of HIV infection/AIDS (e.g., “black spots” on their skin). The stories told by these FSWs exemplify such experiences:

Like this guy who came to our room, he tells me, for a short time I will give you K500,000. You look at his penis, and it's almost rotten. You can barely see it. You try to put this penis in a condom and all that comes out is blood and pus, and surely someone in her normal senses would not have sex with such a man.

(28 years old, lower income clients)

…He went ahead and we had live sex [sex without a condom], but before he released, there was blood everywhere. The man was just looking very healthy and he was even wearing a good perfume. He had insisted that he was not sick, but from the look of things… semen is usually white but his was red.

(25 years old, higher income clients)

Several respondents suggested that they could discern if a client was HIV-infected (“sick”) by his appearance; all suggested they would use condoms with men who looked ill. Several also reported that the request for unprotected sex itself was a red flag, as it was assumed that only someone who knew he was HIV-infected would risk live sex with a prostitute.

Condom use was frequent but not consistent. About half of the respondents reported regular condom use with clients although many FSWs told of situations in which condom negotiations were not successful or when they were willing to have live sex. With paying clients, the promise of more money in periods of financial stress was the most common reason for agreeing to unprotected sex, despite acknowledging the risks.

If he has money, I would have sex with him and afterwards go home and take some antibiotics to cleanse myself of any infections that he would have given to me. But there are some clients that look really sick and with those you can't risk not to use a condom.

(32 years old, middle income clients)

Indeed, A few FSWs also reported that when the man was desirable, they would agree to have unprotected sex, as illustrated by this respondent:

Ok, there are times that you look at this man and… like well, this guy looks gorgeous and, not to lie to you, I agree to live sex.

(27 years old, middle income clients)

Half (9 of 20) of the respondents reported never using condoms with non-paying partners. Boyfriends, who were often married to other women, reportedly used a variety of techniques to persuade FSWs to forgo condom use within their relationship, even when her status as a sex worker was known.

For several respondents, feelings of love toward their regular partners and their own sexual desires led them to engage in unprotected sex, even while acknowledging the risks.

### Male Circumcision: FSWs understanding of risk and experience with circumcised clients

Descriptions of sexual encounters with paying clients and non-paying partners suggest the multiple challenges faced regularly by FSWs to engage in safer sex. It is into this context of sex that information about male circumcision is interpreted, and the context in which FSWs encounter circumcised men. In the sections that follow we explore how respondents have gained information on MC, how they understand and misunderstand MC's protective effects, and their experience with circumcised clients.

### Sources of Information Regarding MC

Respondents' knowledge regarding MC was gained through a variety of sources, including conversations with family, friends, community members, and the clients themselves. For three FSWs, all from Zimbabwe, clients were their only source of information. A few FSWs reported hearing something about MC on television or the radio; one recalled hearing news stories of parents bringing their sons to clinics to be circumcised, and two reported that while they had heard mention of MC in the media, they could not remember what was said as they were not paying close attention. Two reported seeing posters or stickers about MC in a clinic setting, and one was advised of the benefits of MC when she gave birth to her son (she declined to have him circumcised). Finally, one FSW reported that she had heard about MC from outreach workers distributing condoms. Thus, while most respondents had heard of MC from multiple sources, the majority had not received comprehensive information on MC from a reliable source.

### Female sex workers' understanding of male circumcision

All of the respondents had heard of MC and could define it, when prompted, as the removal of the foreskin. At the same time however, nearly all of the respondents indicated that their understanding of MC was not complete. For example, when asked for elaboration, or when prompted to provide more details, respondents replied with phrases such as “nothing else, that is all” (21 years old, lower income clients) or “That is all I heard” (34 years old, middle income clients). The relatively superficial information on MC received contributed to how FSWs understood the information, and how they reconciled it with their existing – largely accurate – knowledge of how HIV is transmitted and their sexual experiences with circumcised men. The result was that respondents were largely skeptical of MC's ability to protect against HIV, but believed some benefits to MC were plausible, and others pleasurable. Their understanding of specific aspects of MC, including potentially protective effects for men and women, are presented below.

A widely-held view that emerged from the interviews was the depiction of the foreskin as “dirty,” and a place where diseases are harbored; therefore, its removal makes a man “clean” and less likely to get “sick.” Many shared the idea that circumcision allows a man to more thoroughly clean his genitalia after sex, thus washing away infections and reducing the chances of disease acquisition. Similarly, since circumcised men were seen as cleaner, some FSWs expressed the belief that they were less likely to transmit infections to sexual partners after exposure, as an infection could not survive on the penis without a foreskin. Others, however, noted that infections could also be passed via semen, thus a condom offered greater protection than circumcision.

Indeed, many FSWs (11 of 20) expressed doubt that MC offered any protective effects for women, a few believed it offered protection against STIs and one believed it protected against HIV (the remainder of responses were not clear). FSWs who did not believe that MC protects women typically responded as follows:

I: For women, is MC more, less, or as effective as condoms in protecting against HIV infection?

R: Women should continue to use condoms to protect themselves other than saying ‘my partner is circumcised hence I can't get any infection.’ A condom should still be used.

I: When did you understand this?

R: Just from my own thoughts, nobody told me about it.

(27 years old, middle income clients)

I: If a man is circumcised does it protect his female sex partners from getting HIV?

R: If he doesn't use a condom you can get HIV whether circumcised or not.

I: Is MC more, less, or as effective as condoms in protecting against HIV infection?

R: A condom should ever be there – that's the one I know protects.

(35 years old, middle income clients)

There was some confusion in regard to the protection MC offered for men. The concept of partial efficacy of MC was not well understood by the respondents; rather, the most common understanding of MC was that it was fully effective in preventing the transmission of STIs, but not effective for reducing the transmission of HIV from women to men. This was articulated in many similar ways by the respondents:

They say because they are circumcised, they can't get sick, yet it's not really true. They are only protected from STIs.

(23 years old, middle income clients)

I: Does MC protect a man from getting HIV?

R: No, he can still contract HIV, maybe he is protected from small, small diseases like syphilis and other STIs.

(32 yrs. old, middle income clients)

As such, MC was not widely believed to be an HIV prevention strategy, but was viewed by some (7 of 20) as preventive against STIs for men. There was no class distinction evident in the data; sex workers who had lower, middle, and higher income clients expressed this belief.

Five of the FSWs believed that MC was protective for men against STI and HIV transmission, but each offered caveats. One respondent suggested that MC is protective only if the man does not have a cut on his penis. For two respondents, “dry sex” could still result in HIV infection. As one FSW explained:

I: Can a man still get HIV if he has been circumcised?

R: Yes, he can get it.

I: In which way?

R: Through dry sex.

I: What if it's wet sex and no bruises, can one still get HIV?

R: No he can't.

(34 years old, middle income clients)

In the absence of a full understanding of MC, some respondents used their understanding of other HIV prevention and treatment efforts to inform their opinions of MC. For example, one FSW used anecdotal information about antiretroviral (ARV) therapy to speculate what the protective effect of MC might be:

I wouldn't really know because I don't know the truth behind all this… I think on another angle I can believe it because just like they talk about ARVs, they say if you take them, you gain weight, and people gain for real, so it's the same with circumcision. [When] people say they can't get diseases, it could be true.

(25 years old, higher income clients)

Similarly some relied on information about general HIV transmission in formulating their beliefs about MC efficacy. As one FSW explains:

I don't believe that [MC is protective] because diseases are found in the blood, not because someone is circumcised.

(27 years old, middle income clients)

For others, incomplete or conflicting information lead them to discount the idea of MC as protective against either/both STIs and HIV. Notably, the three respondents who had received information about MC only from the clients themselves expressed the clearest doubt in regard to MC's potentially protective effects.

Despite these uncertainties or skepticism, however, many FSWs expressed a preference for circumcised sexual partners. This respondent's comments were representative of those who suggested that circumcised men were cleaner, “sweeter” (a term having to do with pleasure), and better lovers (as they were slower to ejaculate).

Those that are circumcised are sweet. Even if they wear a condom, it just feels like it is live [unprotected].

(23 years old, middle income clients)

Although the respondents expressed a preference for circumcised clients, there was no price difference between what FSWs charged men who were circumcised for sex, and what they charged men who were not circumcised.

### Condom Negotiations and Use with Circumcised Clients

Although almost all respondents had incomplete information about MC, nearly all reported that a condom is the best available protection from HIV transmission, even if they break (either by accident or as the result of sabotage) or are not used for other reasons.

I: Do you believe that circumcision protects a man from getting HIV?

R: Not even a bit, I do not believe that.

I: Have you heard that circumcision gives more protection, about the same amount of protection or less protection than the condom?

R: Condoms give more protection.

I: Why?

R: Because that man will release his sperm in the condom and also my vaginal fluids will end up just around the condom, so the one who is circumcised is not safe if he's not wearing a condom.

(30 years old, high income clients)

All of the FSWs in the study had experience with circumcised clients. For two respondents, this experience was limited to one client, however for many of the FSWs interviewed, in their estimations, at least half of their clients were circumcised. Many reported that the number of MC clients was continuously increasing. While most men, regardless of circumcision status, tried to negotiate unprotected or “live” sex, as previously noted, circumcised clients used their circumcision status as a bargaining tool to convince FSWs to have unprotected sex. The following quotes suggest the process:

One who is circumcised will argue with you for hours that his penis is clean and you can't get diseases.

(30 years old, higher income clients)

They [circumcised men] do insist on live sex, yes. And they say that they are clean and can't get diseases like STIs. Though I have not had a lot of circumcised men, at least those I have had insisted on live sex.

(23 years old, middle income clients)

Okay, let me say a lot of people are not yet educated about this circumcision thing. They think once you are circumcised you are a free man and you can't get sick. Okay they have got that part all wrong, most of them wouldn't want to use a condom.

(21 years old, higher income clients)

One respondent's comments illustrate the role of alcohol in jeopardizing condom use:

I: Do you sometimes give the circumcised men the benefit of doubt that you can't get diseases from them if they ask for live sex?

R: Yes, … sometimes we get drunk and when you look at a man, just the way he looks, sometimes we lose our senses and have live sex with that man.

(27 years old, middle income clients)

Some sex workers in the study suggested that circumcised men took other sexual risks as a result of their MC. As one FSW explained:

I think circumcision is just making men become stupid when it comes to issues of sex… These men are supposed to be told the risks of having live sex, even when they are circumcised, but it looks like they are told that they are safe now– they think that they can screw anybody they want.

(30 years old, higher income clients)

### Sex with Recently Circumcised Clients

Three of the twenty FSWs interviewed reported having clients who were very recently circumcised:

…I think it was recent because the tip of his penis looked red. I even asked him if he was circumcised recently and he said yes… I didn't ask him [when], but I think he was well healed.

(27 years old, middle income clients)

I: The one who came with the stitches, was the wound still looking fresh?

R: Yes, it was.

I: Like he had undergone circumcision how many days ago?

R: He told me by then it was like two weeks ago… He said his wife refused to have sex with him, and that is why he came looking for a sex worker.

(25 years old, higher income clients)

In recalling these experiences, the FSWs again highlighted the impact of poverty on sexual decision making:

R: As an adult, some usually have stitches on the penis.

I: That is before the stitches fall off – before they are completely healed?

R: Yes, before they are completely healed.

I: Don't you get scared that maybe the wound can bleed or something?

R: We do, but you just have to be strong because you need the money.

(21 years old, lower income clients)

I: Didn't you mind having sex with him if he had stiches?

R: No, because all I want is the money.

‘I: When you were having sex with him did he seem to enjoy it, or was it painful?

R: I think it was painful because after the act there was lots of blood in the condom.

(25 years old, higher income clients)

All reporting using condoms with these clients, although it required some negotiation.

## Discussion

Zambia has a somewhat higher prevalence of sex worker encounters than is reported in most African countries [22]. Males start visiting sex workers at an early age −10% of sexually active males aged 15–16 ever paid for sex with the proportion rising up to 26% of males aged 25. Zambian men who visit sex workers have other characteristics that increase the likelihood of engaging in riskier sex, such as alcohol abuse, and approval of gender based violence. As in many other settings, earlier in the country's epidemic, sex with commercial sex workers played a larger role in HIV transmission. Leclerc and colleagues constructed a simulation model based on HIV prevalence in 2001 that indicates that over a third (36.4%) of male infections occurred to men during contact with commercial sex workers [23]. As the epidemic in Zambia has become generalized to the heterosexual population, sex workers play a less significant, but still notable, role in HIV transmission. UNAIDS indicates that in 2008, 6.5% of new infections were related to FSWs – while a decrease from the previous decade, commercial sex work remains one of the top four sources of new infections in Zambia [18]. Sex workers are thus a vital population to address in MC programs in Zambia – both for the safety and health of sex workers, and the health of their clients.

Previous research has shown that the lives of FSWs in sub-Saharan Africa frequently include interpersonal violence, police harassment, and alcohol and drug use [14], [24]–[27]. Our data concur, highlighting the alcohol use, violence, and unprotected sex with sexual partners that increase FSWs' vulnerability to HIV infection. Most of our sample had multiple concurrent partners, echoing findings from previous studies of FSWs in sub-Saharan Africa [28], and Zambia [20]. The fluidity between paying clients, regular clients, and casual partners was apparent in the accounts of the FSWs, and has been noted in other studies of FSWs in Africa [24]. This fluidity complicates whether and how consistently condoms are used. A survey of sex workers in four sites in Zambia found that only 29% of respondents reported using a condom every time with non-paying partners in the past year [20]. These data are consistent with our findings. Low condom use with nonpaying partners and concurrencies creates a social network of high risk between clients, their wives, FSWs, their sex partners, and their sex partners' wives.

This exploratory study in Lusaka, Zambia examined female sex workers' understanding of MC, their experiences with circumcised and uncircumcised clients, and their risk of HIV/STI. In assessing the impact of MC on a most-at-risk population, our results suggest that FSWs in Zambia, already at high risk for HIV infection, are affected by the scale-up of MC services, either because clients are seeking out FSWs before their wounds are healed, or because condom negotiations, an already challenging aspect of doing sex work, are being further complicated as clients attempt to use their MC status to procure unprotected sex. Fortunately however, at the time of this study, most of the FSWs reported skepticism about circumcised men's claims of being “clean” and most FSWs reported that condoms were a better prevention method and used them on a somewhat regular basis with clients.

The data also suggest that the educational and community awareness efforts surrounding MC scale up efforts in Zambia had not yet reached the FSW population. Typically, FSWs' information on MC was garnered from peers or clients; only a few of the FSWs had received information about MC directly from a health care provider or HIV prevention (condom) outreach worker. Most FSWs in the sample believed MC could protect men against STIs, but not HIV. Additionally, most thought – accurately – that MC offered no protective effects for women, but, notably, were not certain of this and most had deduced this themselves.

While FSWs routinely have to negotiate condom use with clients regardless of circumcision status, and face economic pressures not to use condoms, as shown in our data MC has created a new strategy for male clients seeking live sex that could potentially serve to undermine FSWs attempts to use condoms consistently. Thus, as MC scale up efforts are stepped up, an opportunity exists to support FSWs efforts to protect themselves by providing full and accurate information about what MC can – and cannot – offer for HIV infection prevention, particularly for women.

There were several limitations to this study. While interviews went well and participants spoke easily about their experiences, evident by most staying longer to chat with the interviewers, participants may have been reluctant to report stigmatized behaviors and/or been vulnerable to social desirability bias. Generalizability to other African FSWs may be limited. Our respondents, drawn from convenience sampling, were easily-identified FSWs engaged in formal sex work in an urban setting. It is unknown if FSWs in rural areas, and/or women who are engaged in informal sex work or transactional sex, are exposed to similar risks. Moreover, our sample may under represent the most vulnerable FSWs who may be less willing to participate in an interview or be harder to reach.

Despite the small scope of this study, we offer several programmatic recommendations. First, as MC services are scaled up in sub-Saharan Africa, it is critical that all women, including FSWs, be provided with accurate information about MC, including the limits of its protective effect for men and lack of protection for individual women, as well as the importance of post-surgical abstinence. Second, MC providers should reach out to organizations that offer services and support to FSWs to ensure that FSWs receive accurate information about MC. Third, demand creation information campaigns should, at a minimum, ensure that no misleading information or images are conveyed to women in an effort to enhance demand efforts. When message platforms allow for more detailed information, they should use the opportunity to more comprehensively inform both men and women about MC and HIV. Fourth, information sources that may filter to FSWs – such as HIV prevention counseling provided at VCT clinics – should also be clear and direct about the limits of MC's protective effects and the importance of refraining from sex during wound healing. Finally, it is imperative that MC counseling intensify efforts to impress upon men – particularly those men who have riskier sex [29] –the importance of refraining from sex during wound healing, as well as the need for continued use of condoms. By including consideration of FSWs in a national MC strategy to decrease HIV prevalence, we can support a vulnerable and stigmatized population, strategically counter men predisposed to engage in risk compensation, target sexual network nodes that are at very high risk even before MC-related risk compensation, and thus hopefully increase the effectiveness of national HIV prevention efforts.
